# Effect of densely ionizing radiation on cardiomyocyte differentiation from human‐induced pluripotent stem cells

**DOI:** 10.14814/phy2.13308

**Published:** 2017-08-11

**Authors:** Erdene Baljinnyam, Sundararajan Venkatesh, Richard Gordan, Satvik Mareedu, Jianyi Zhang, Lai‐Hua Xie, Edouard I. Azzam, Carolyn K. Suzuki, Diego Fraidenraich

**Affiliations:** ^1^ Department of Cell Biology and Molecular Medicine New Jersey Medical School Rutgers Biomedical and Health Sciences Newark New Jersey; ^2^ Department of Microbiology, Biochemistry and Molecular Genetics New Jersey Medical School Rutgers Biomedical and Health Sciences Newark New Jersey; ^3^ Department of Biomedical Engineering School of Medicine and School of Engineering University of Alabama at Birmingham Birmingham Alabama; ^4^ Department of Radiology New Jersey Medical School Rutgers Biomedical and Health Sciences Newark New Jersey

**Keywords:** Arrhythmogenesis, hiPSC‐CM, ionizing radiation, mitochondria

## Abstract

The process of human cardiac development can be faithfully recapitulated in a culture dish with human pluripotent stem cells, where the impact of environmental stressors can be evaluated. The consequences of ionizing radiation exposure on human cardiac differentiation are largely unknown. In this study, human‐induced pluripotent stem cell cultures (hiPSCs) were subjected to an external beam of 3.7 MeV *α*‐particles at low mean absorbed doses of 0.5, 3, and 10 cGy. Subsequently, the hiPSCs were differentiated into beating cardiac myocytes (hiPSC‐CMs). Pluripotent and cardiac markers and morphology did not reveal differences between the irradiated and nonirradiated groups. While cell number was not affected during CM differentiation, cell number of differentiated CMs was severely reduced by ionizing radiation in a dose‐responsive manner. *β*‐adrenergic stimulation causes calcium (Ca^2+^) overload and oxidative stress. Although no significant increase in Ca^2+^ transient amplitude was observed in any group after treatment with 1 *μ*mol/L isoproterenol, the incidence of spontaneous Ca^2+^ waves/releases was more frequent in hiPSC‐CMs of the irradiated groups, indicating arrhythmogenic activities at the single cell level. Increased transcript expression of mitochondrial biomarkers (LONP1, TFAM) and mtDNA‐encoded genes (MT‐CYB, MT‐RNR1) was detected upon differentiation of hiPSC‐CMs suggesting increased organelle biogenesis. Exposure of hiPSC‐CM cultures to 10 cGy significantly upregulated MT‐CYB and MT‐RNR1 expression, which may reflect an adaptive response to ionizing radiation. Our results indicate that important aspects of differentiation of hiPSCs into cardiac myocytes may be affected by low fluences of densely ionizing radiations such as *α*‐particles.

## Introduction

The molecular series of events that lead to the development of a heart are primarily governed by developmental cues (Srivastava 2006; Chien et al. 2008; Kuyumcu‐Martinez and Bressan 2016). This process can be faithfully recapitulated in a culture dish, with pluripotent stem cells as starting material (Spater et al. 2014; Burridge et al. 2015). The in vitro platform of cardiac differentiation allows investigators to evaluate potentially detrimental consequences of changes in the environment, such as ionizing radiation.

Humans are constantly exposed to low‐level ionizing radiation from natural sources. Furthermore, normal tissue may be exposed to low doses/low fluences of radiation during the course of occupational activities or radiotherapy (Azzam et al. 2003, 2016). The use of different irradiation modalities remains a widely used means to treat pediatric cancers as well as other pathological conditions. In the course of these treatments, high doses are targeted to the affected area. However, normal tissue (i.e., heart) may also be affected as a result of exposure to scatter radiation or to the spread of harmful effects from irradiated tissue to bystander normal tissue in the vicinity (Newhauser and Durante 2011). An understanding of the biological changes occurring in the normal tissue as a result of low‐level exposures or due to bystander effects is critical to estimating health risks and to devising strategies to avoid adverse long‐term effects.

The purpose of this study was to evaluate the effects of ionizing radiation exposure on cardiac differentiation. To achieve this, we developed a system whereby human‐induced pluripotent stem cells (hiPSCs) maintained in culture are exposed to low fluences of *α*‐particles. HiPSCs from the irradiated cultures were then differentiated into beating cardiomyocytes (hiPSC‐CMs), and molecular, morphological, and functional assessments were conducted. We report that low mean absorbed doses of *α*‐particles applied to hiPSCs does not affect their capacity to become beating cardiomyocytes, but has direct consequences on spontaneous Ca^2+^ beating and number of the differentiated cardiomyocytes obtained.

## Materials and Methods

### Cells

A hiPSC line derived from human cardiac fibroblasts was generated in the laboratory of Zhang et al. (2015). These cells contain a GFP reporter gene under the cardiac promoter sequences of *α*MHC (Zhang et al. 2015). HiPSCs were maintained in mTESR1 medium (STEMCELL Technologies), in six‐well plates coated with hESC‐qualified Matrigel (BD Biosciences) in the presence 5 μmol/L ROCK inhibitor (Y‐27632) to ensure optimal attachment. hiPSCs were cultured in clusters to avoid spontaneous differentiation. Cells were passaged at 1:36 dilution in six‐well plates every 5–7 days. They were disaggregated with Versene (Life Technologies). Under these nondifferentiating conditions, hiPSCs express pluripotent markers TRA‐1‐60, Oct3/4, and hNanog (Figure S1).

### Alpha‐particle irradiation

HiPSCs grown for 3–4 days were dissociated and seeded in stainless steel dishes with a circular 36‐mm diameter growing surface that consists of 1.5‐*μ*m‐thick replaceable polyethylene terephthalate (PET) (known as Mylar). To facilitate cell attachment, the PET surface was precoated with Matrigel solution, overlaid with 2 mL of mTESR1, and incubated at 37°C overnight. Mylar membranes were coated with Matrigel for 1 h. Cells originating from one well of a six‐well plate containing hiPSCs were seeded into 4 Mylar dishes with equal number of cells for control (nonirradiated sham), 0.5 cGy, 3 cGy, and 10 cGy treatments and incubated for 24 hours. Alpha‐particle irradiations were conducted with a 7.4‐MBq ^241^Am collimated source housed in a helium‐filled Plexiglas box located in a chamber at 37°C with an atmosphere of 5% CO_2_ (vol/vol) in air. To optimize uniformity of the beam, the source was mounted on a rotating platform (88 rpm) and the exit window was equipped with a beam delimiter. The uniformity was confirmed by etching PADC plastic exposed to the beam for 4 sec. Cells were irradiated at a mean absorbed dose rate of 2 cGy/min, and irradiation of samples occurred from below through the PET growing surface. At the latter surface, *α*‐particles had a measured mean energy of 3.7 MeV (0.92 MeV/u) with full width at half maximum (FWHM) of 0.5 MeV. The linear energy transfer (LET) corresponding to a mean energy of 3.7 MeV is ~109 keV/*μ*m in liquid water. The irradiator box was fitted with a photographic shutter to allow accurate delivery of the desired mean absorbed dose (Neti et al. 2004). The hiPSC cells were grown in the presence of 10 μmol/L ROCK inhibitor Y‐27632 for 3–4 days to ensure good attachment. The cells were subjected to the *α*‐particles at mean absorbed doses of 0 (sham), 0.5, 3, and 10 cGy. The energy lost by the *α*‐particle is essentially local and not transported beyond the cell being traversed. At these mean absorbed doses, a large fraction of the cells in the population are not traversed through the nucleus by an *α*‐particle track (~3% of nuclei are estimated to be traversed at 0.5 cGy, ~15% at 3 cGy, and ~50% at 10 cGy). Thirty minutes after irradiation, the hiPSCs were harvested and transferred to a Matrigel‐coated six‐well plates followed by 12 days of GiWi cardiomyocyte differentiation (CD).

### Cardiomyocyte differentiation

**Figure 1:**
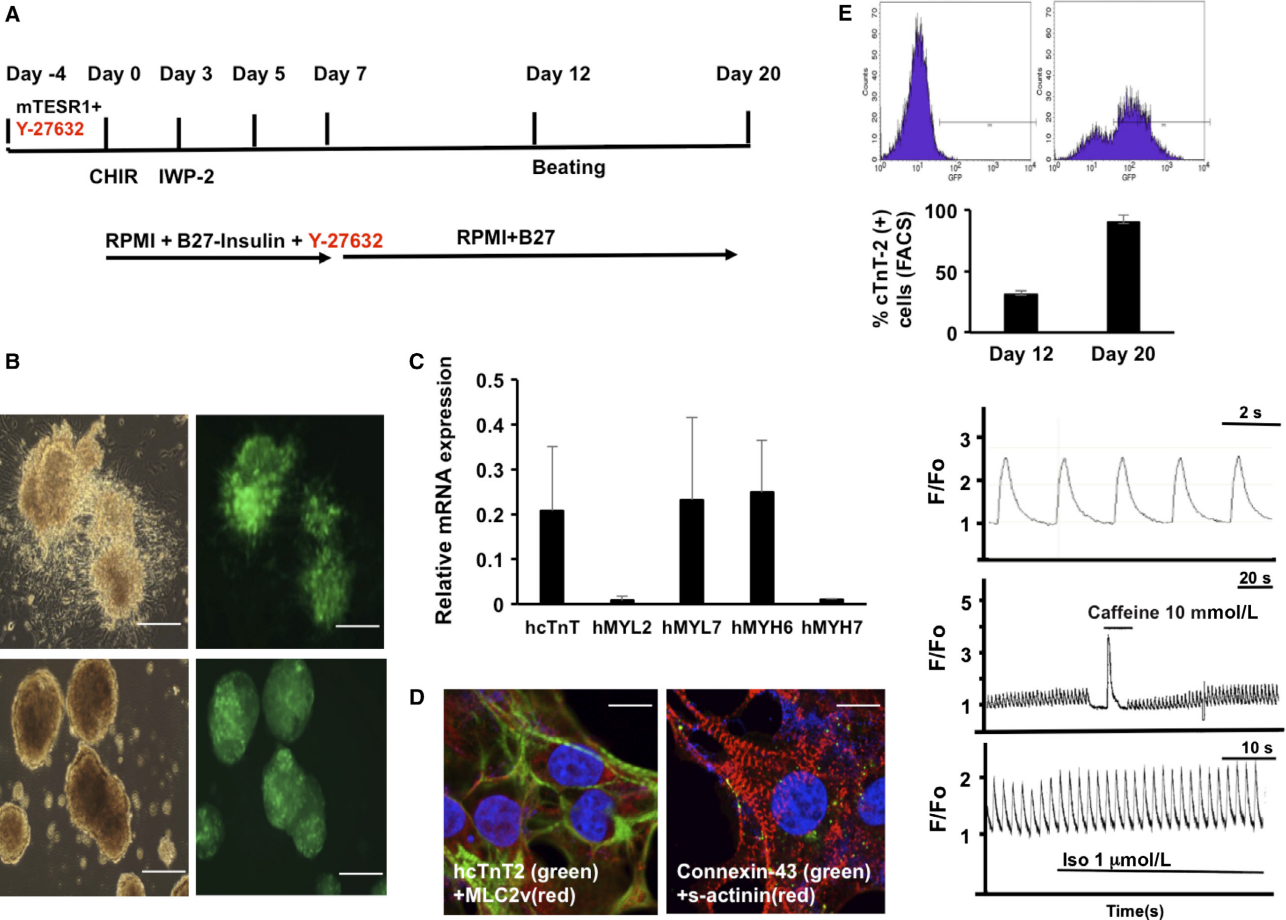
Analysis of hiPSC‐CMs. (A) Schema of the GiWi cardiac differentiation protocol modified with Y‐27632 additions (in red). (B) Spheroids of hiPSC‐CMs expressing *α* MHC‐eGFP at days 12 (top) and 20 (bottom) of differentiation. Left, bright field; right, GFP signal. Scale bar = 100 *μ*m. (C) QRT‐PCR analysis of hiPSC‐CM contractile markers; human adult heart mRNA expression = 1. (D) Immunofluorescence staining of contractile markers (cTnT2, MLC2v, and cardiac s‐actinin) and connexin‐43 in hiPSC at day 20 of differentiation. Scale bar = 10 *μ*m. (E) Top: GFP‐based FACS analysis of *α* MHC‐GFP‐hiPSC‐CMs (right). Note that *α* MHC does not drive GFP expression in undifferentiated hiPSCs (left). Bottom: Percentage of the cTnT‐2‐positive hiPSC‐CMs at days 12 and 20 of differentiation assessed by FACS analysis; *n* = 3. (F) Calcium transient measurements in hiPSC‐CMs; top: spontaneously beating hiPSC‐CMs, *T*_50_ = 300 ms; middle: caffeine 10 mmol/L stimulation, indicating SR Ca release; bottom: isoproterenol stimulation and pacing.

Cardiomyocyte differentiation was carried out using a published method employing the GSK inhibitor CHIR99021 and a Wnt inhibitor IWP‐2 (GiWi protocol) (Lian et al. 2013), modified with the addition of ROCK‐specific inhibitor Y‐27632 (10 *μ*mol/L) until day 7 (Fig. 1A). Briefly, hiPSCs were cultured on Matrigel‐coated six‐well plates in mTeSR1 medium. Cells were resuspended in mTeSR1 with the ROCK‐specific inhibitor Y27632 (Tocris) (10 *μ*mol/L) and plated at varying cell densities. On day 0, when the cells reached 50–60% confluency, the medium was aspirated and the cells were incubated in RPMI/B27 (Life Technologies) without insulin containing the GSK inhibitor CHIR99021 (10 *μ*mol/L). On day 3, the cells were treated with Wnt inhibitor IWP2 (5 *μ*mol/L) in RPMI/B27 without insulin. From day 7 onward, the cells were cultured in RPMI/B27 with insulin. Under these optimized conditions, >90% of purity (as determined by FACS analysis) was achieved at days 12 and 20 (Fig. 1E). Similarly, >90% of purity (FACS analysis) was achieved at days 12 and 20 with human embryonic stem cells (hESCs, H7, WiCell) and with dermal fibroblasts reprogrammed hiPSCs (Coriell Institute, GM23338). CHIR99021 was purchased from Selleck Chemicals. ROCK inhibitor Y‐27632 and Wnt inhibitor IWP‐2 were from Tocris. All other media, materials, and reagents were from Fisher Scientific/Invitrogen unless specified.

### Cell proliferation assay

Cells were plated at the density of 4000 or 8000 (differentiated or differentiating) cells, respectively, per well into a 96‐well plate (*n* = 8 for each group), and cultured for 24 hours. Ten microliter of yellow tetrazolium MTT reagent (3‐(4, 5‐dimethylthiazolyl‐2)‐2, 5‐diphenyltetrazolium bromide, ATCC) was added to each well and incubated for 2 hours until a purple precipitate was visible. Hundred microliter of detergent reagent was added to each well and left at room temperature in the dark for 2 hours. Absorbance was determined at 570 nm, and normalized to that of control groups.

### Quantitative RT‐PCR

After day 12 of irradiation, the presence of cardiac markers was determined by quantitative RT‐PCR (QRT‐PCR). Total RNA was extracted and treated with DNAseI and with an RNeasy mini kit (Qiagen) and converted into cDNA using the TaqMan RT kit (Applied Biosystems). QRT‐PCR was performed with the QuantiTect SYBR Green PCR Kit (Qiagen) according to the manufacturer's recommendations on a Biorad CFX96 real‐time PCR machine. Primers for hBrachyury, hMESP1, hNkx2.5, hcTnT‐2, contractile markers such as human myosin light chain hMYL2, hMYL7, myosin heavy chain hMYH6, hMYH7, the fibroblast marker hTHY1, and the endothelial marker hPECAM were from IDT. Relative expression levels were compared to those obtained from mRNA from human hearts (Ambion). Expression of markers was normalized to 18S ribosomal RNA. Primer sequences are listed below:

hcTnT‐2: F‐TTCACCAAAGATCTGCTCCTCGCT, R‐TTATTACTGGTGTGGAGTGGGTGTGG

hBRACHYURY: F‐TGTCCCAGGTGGCTTACACATGAA, R‐GGTGTGCCAAAGTTGCCAATACAC

hMESP1: F‐AGCCCAAGTGACAAGGGACAACT, R‐AAGGAACCACTTCGAAGGTGCTGA

hNKX2‐5: F‐TTTGCATTCACTCCTGCGGAGACCTA, R‐ACTCATTGCACGCTGCATAATCGC

hMYH6: F‐TCAGCTGGAGGCCAAAGTAAAGGA, R‐TTCTTGAGCTCTGAGCACTCGTCT

hMYH7: F‐TCGTGCCTGATGACAAACAGGAGT, R‐ATACTCGGTCTCGGCAGTGACTTT

hMYL2: F‐TGTCCCTACCTTGTCTGTTAGCCA, R‐ATTGGAACATGGCCTCTGGATGGA

hMYL7: F‐ACATCATCACCCATGGAGACGAGA, R‐GCAACAGAGTTTATTGAGGTGCCC

hPECAM: F‐TTCCTGACAGTGTCTTGAGTGGGT, R‐TTTGGCTAGGCGTGGTTCTCATCT

hTHY1: F‐ATACCAGCAGTTCACCCATCCAGT, R‐ATTTGCTGGTGAAGTTGGTTCGGG

### Immunofluorescence

HiPSC were cultured on Matrigel‐coated cover slips in mTeSR1 for 2 days. Immunofluorescent staining for OCT3/4 (Santa Cruz Biotechnologies, 1:200), hNANOG (R&D systems, 1:200), and Alexa Fluor‐488 anti‐human TRA‐1‐60‐R (BioLegend, 1:50) were performed on undifferentiated hiPSC. Standard immunofluorescence staining was performed to confirm the expression and distribution of cardiomyocyte markers using antibodies reactive with cardiac troponin T (hcTNT‐2, Abcam, 1:50), s‐Actinin (Sigma/A7811/Clone: EA‐53 1:500), myosin light chain 2v (MLC2v, Synaptic Systems, 1:100), connexin‐43 (Sigma, 1:200). The secondary antibodies used were Alexa 488 goat anti‐rabbit IgG/A‐11008 (Invitrogen, 1:400) and Alexa 594 goat anti‐mouse IgG2b/A‐21145 (Invitrogen, 1:400).

### Confocal microscopy

HiPSC cultured on Matrigel‐coated glass bottom chambers (Maltek) were stained for immunofluorescence and subjected to confocal imaging to visualize sarcomere structure using the Nikon A1RSI confocal microscope equipped with a 20× Plan Apo VC objective lens. The confocal images were analyzed using Nikon NIS elements software.

### Flow cytometry

To determine the purity of hiPSC‐derived cardiomyocytes, flow cytometry analysis of hiPSC‐derived cardiomyocytes was performed as described previously (Lian et al. 2013). At days 12 and 20 of differentiation, the cells were washed with PBS, treated with Accutase (Innovative Cell Technology), and dissociated. After counting the cells with a hemocytometer, the cells were centrifuged at 200*g* for 5 min and the pellet fixed with 4% paraformaldehyde. The fixed cells were treated with 1 mL of 90% cold methanol. The cells were washed with FlowBuffer‐1 (0.5% BSA in PBS) and resuspended in 100 *μ*L of FlowBuffer‐2 (0.1% Triton X and 0.5% BSA in PBS) with the 1:400 dilution of primary antibody for hcTnT‐2 (Abcam) and incubated overnight at 4°C. After washing the cells with FlowBuffer‐2, the cells were incubated for 30 min at room temperature in the dark, followed by serial washes with FlowBuffer‐2. The cell pellet was resuspended in 300 *μ*L FlowBuffer‐1 and transferred into flow round‐bottom tubes placed on ice for the flow cytometric analysis with a FACS Calibur. FL1 channel was utilized to analyze for GFP/FITC signal. Live FACS analysis for *α*MHC‐eGFP‐positive cells was done in a similar way without cell fixation and antibody staining in FlowBuffer‐2.

### Measurement of Ca^2+^ transients

Cells were loaded with the Ca^2+^ indicator Fluo‐4 AM (5 *μ*mol/L for 40 min). Ca^2+^ fluorescence (EX/EM: 485/530 nm) was monitored using a Nikon Eclipse TE200‐inverted microscope and recorded using an Ixon charge‐coupled device camera (Andor Technology) operating at ~50 fps, as described in our previous studies (Zhao et al. 2013, 2015). The fluorescence intensity was measured as the ratio of fluorescence (*F*) over basal diastolic fluorescence (*F*_0_).

### Mitochondrial stress markers

Using quantitative PCR (qPCR), we measured the transcript levels of Lon and ClpP – mitochondrial ATP‐dependent proteases, Tid1 – a mitochondrial chaperone, mitochondrial transcription factor A (TFAM) – the major regulator of mitochondrial DNA (mtDNA) transcription, replication and segregation, and mtDNA‐encoded cytochrome b (MT‐CYB) and mtDNA‐encoded 12S ribosomal RNA (MT‐RNR1). The cells were pelleted, washed one time with PBS, and total RNA was isolated using Qiagen RNeasy kit as per the manufacturer's instructions. RNA (500 ng) was converted into cDNA by using high‐capacity cDNA conversion kit (Applied Biosystem). The cDNA (50 ng) was used to analyze the transcript levels of nuclear DNA‐encoded Lon and TFAM, CLPP, TID1, and mtDNA‐encoded MT‐CYB and MT‐RNR1 by using respective human TaqMan gene expression assays (LONP1‐Hs00998404_m1, TFAM‐Hs00273372_s1, CLPP‐Hs00195655_m1, TID1‐Hs00170600_m1, and MT‐CYB‐Hs02596867_s1 and MT‐RNR1‐Hs02596859_g1). Hypoxanthine phosphoribosyltransferase 1 (HPRT1) (Hs02800695_m1) was used as an endogenous normalizing control. The assay was performed in Bio‐Rad CFX96 real‐time PCR machine. The relative quantification of the expression was calculated using ΔΔCt method by the Bio‐Rad CFX96 software.

### Statistical analysis

The statistical difference was calculated by one‐way ANOVA followed by Dunnett's multiple comparisons using GraphPad Instat software. All data were expressed as the mean ± SEM. *P* < 0.05 is considered as significant**.**

## Results

### Characterization of hiPSC‐CMs

As a starting point, we optimized the differentiation process of hiPSCs, using an established protocol of cardiac differentiation, termed GiWi (GSK inhibitor, Wnt inhibitor) (Lian et al. 2013).

The hiPSCs were originally obtained from human cardiac fibroblasts (Zhang et al. 2015). These cells contain a GFP reporter gene, under cardiac promoter sequences (Zhang et al. 2015). Using this protocol, the hiPSCs started beating at day 12 ± 2 of differentiation. From day 7 and onward of CD, the medium contained insulin according to the GiWi protocol and was changed every other day before the cells started beating, after which the medium was changed daily once the hiPSC‐CMs started beating. The beating hiPSC‐CMs showed a tendency to detach from the Matrigel‐coated plate and to organize into spheric formations in suspension that we termed “cardiospheres” under GiWi differentiating conditions (Fig. 1A and B) (Lian et al. 2013). However, modification of the protocol by addition of the Rock inhibitor Y‐27632 during the early phase of differentiation (until day 7) prevented detachment of the differentiating cells (preventing cardiosphere formation), as they remained attached to the Matrigel surface (Fig. 1A). Differentiation conditions were validated using two additional sources of human PSCs (hESCs and dermal fibroblast‐reprogrammed hiPSCs) (data not shown).

**Figure 2:**
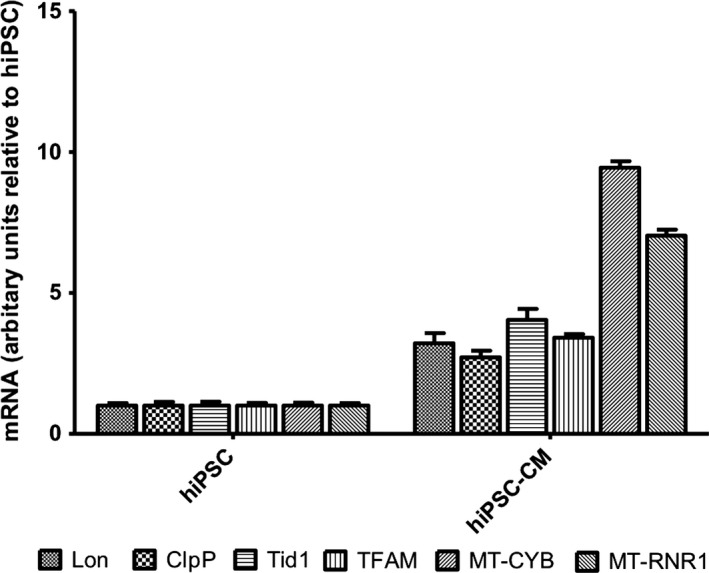
Transcriptional upregulation of nuclear DNA‐encoded mitochondrial Lon, ClpP, Tid1, and TFAM, as well as mtDNA‐encoded transcripts for MT‐CYB and MT‐RNR1 during cardiomyocyte differentiation. HiPSCs were differentiated into beating hiPSC‐CMs, and harvested at day 14. In all the experiments, hiPSC‐CMs started beating at day 12. Three biologically independent experiments were performed (*n* = 3), and for each transcript that was analyzed, triplicate reactions were performed. Values are expressed as relative fold change ± SEM.

Molecular characterization was performed on beating hiPSC‐CMs to analyze (1) expression of standard cardiac and contractile markers and morphology, (2) calcium transients, and (3) expression of mitochondrial stress proteins and mtDNA‐encoded transcripts. At day 20, beating hiPSC‐CMs revealed mRNA expression of the cardiac and contractile markers hcTnT, hMYL7, and hMYH6 (relative to mRNA from adult human heart) (Fig. 1C), as well as the development of sarcomeric organization shown by immunostaining for cardiac troponin T (hcTnT2) and connexin 43 (Cx43) (Fig. 1D). However, they lacked the rectangular morphology typical of adult cardiomyocytes, and lacked confinement of Cx43 to intercalated disks, which denoted immature cardiac morphology (Fig. 1D). The purity of differentiated CMs ranged from 71.2% (GFP‐positive signal) at day 12 to 89 ± 6% (cTnT2 positive) at day 20 (Fig. 1E). Although the beating hiPSC‐CMs had a morphologically immature appearance, they nevertheless displayed spontaneous and paced calcium transients, responded to isoproterenol and caffeine treatment (Fig. 1F). Further metabolic selection in the absence of glucose at day 20 increased the purity of the beating hiPSC‐CMs to values above 95%. In adult cardiac myocytes, mitochondria occupy ~30–40% of cell volume (Katz 2006). Therefore, we examined the transcript levels in beating hiPSC‐CMs of four important nuclear DNA‐encoded mitochondrial stress response and biogenesis proteins, and two mtDNA‐encoded transcripts required for synthesizing proteins of the electron transport chain (ETC) (Fig. 2). At day 14 of hiPSC‐CM differentiation, QRT‐PCR demonstrated the transcriptional upregulation of nuclear DNA‐encoded mitochondrial Lon (3.2‐fold) – an essential protein quality control protease, which is induced by hypoxia and oxidative and endoplasmic reticulum stress (Bota and Davies 2001; Hori et al. 2002; Fukuda et al. 2007; Venkatesh et al. 2012; Bahat et al. 2015); ClpP (2.7‐fold) – a quality control protease, and Tid1 (4‐fold) – a molecular chaperone, which are mediators of the unfolded protein response in mitochondria (Zhao et al. 2002; Haynes et al. 2007); and mitochondrial transcription factor A (TFAM) (4.5‐fold) – a central regulator of mtDNA maintenance, expression, and transmission that is crucial for cardiac function (Wang et al. 1999) (Fig. 2). In addition, we also observed transcriptional upregulation of mtDNA‐encoded MT‐CYB (9.5‐fold) – a subunit of ETC Complex 3, and MT‐RNR1 (7‐fold), which encodes the 12S RNA required for mitochondrial protein synthesis (Fig. 2).

### Exposure to *α*‐particles leads to reduction in the number of differentiated cells

**Figure 3:**
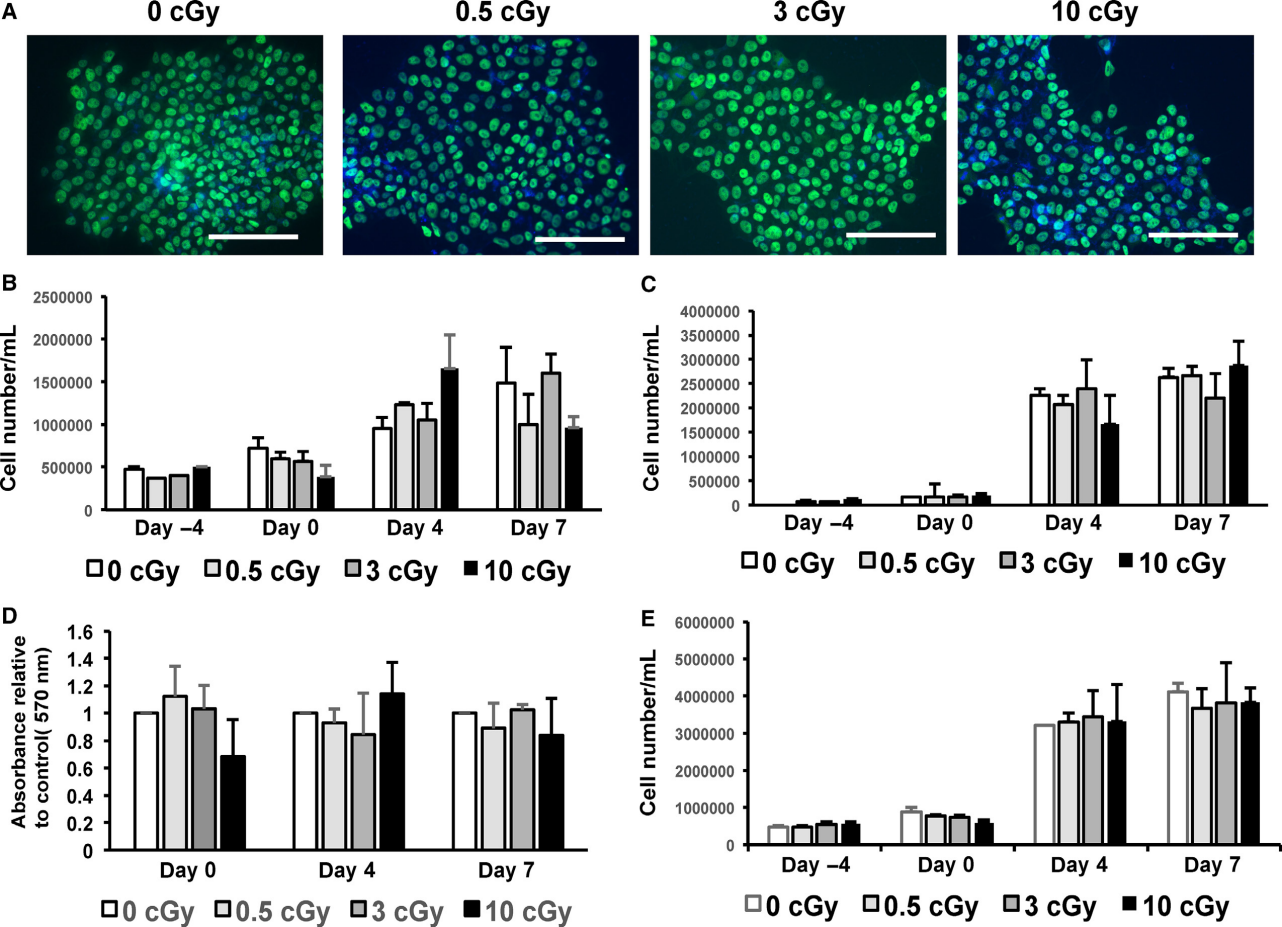
Alpha‐particle irradiation does not affect pluripotency, cell number, cell death, or cell proliferation at early stages of hiPSC differentiation. (A) HiPSCs were irradiated (0.5 cGy, 3 cGy, and 10 cGy), cultured on Matrigel‐coated cover slips in 60 mm dishes for 2 days, and stained for Oct3/4 (green). Blue: DAPI counterstain. Scale bar = 100 *μ*m. (B, C, E) Cell count was performed in nonirradiated and irradiated cells. (B) Number of live cells/mL, (C) number of dead cells/mL, (E) total number of cells counted at days −4, 0, 4, 7 of cardiac differentiation. Three biologically independent experiments were performed (*n* = 3). *P* > 0.05. (D) MTT cell proliferation assay was performed in nonirradiated and irradiated cells at days 0, 4, and 7 of cardiac differentiation. Three biologically independent experiments were performed (*n* = 3). *P* > 0.05.

**Figure 4:**
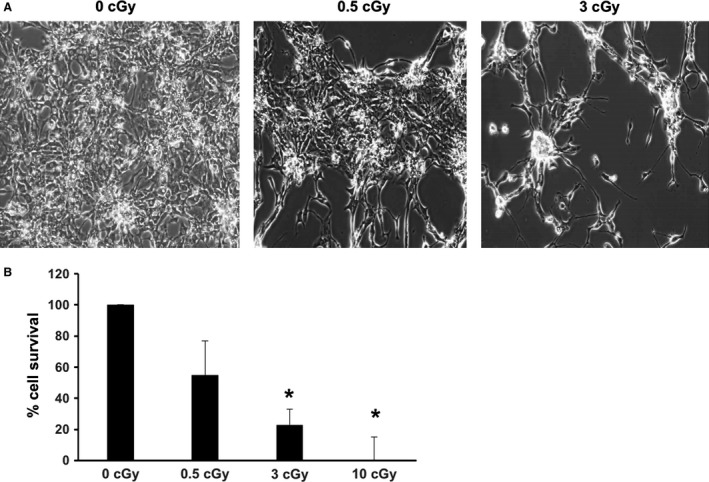
Alpha‐particle irradiation leads to reduction in the number of differentiated cells. (A) Representative images of beating nonirradiated and irradiated (0.5 cGy and 3 cGy) hiPSC‐CMs. 10 cGy treatment led to a negligible amount of beating cells (*n* = 9 per group). (B) Bar graph of A. *P < 0.001. See Videos S1–S3 for visualization of CM beating. Cells were harvested at day 12.

**Figure 5:**
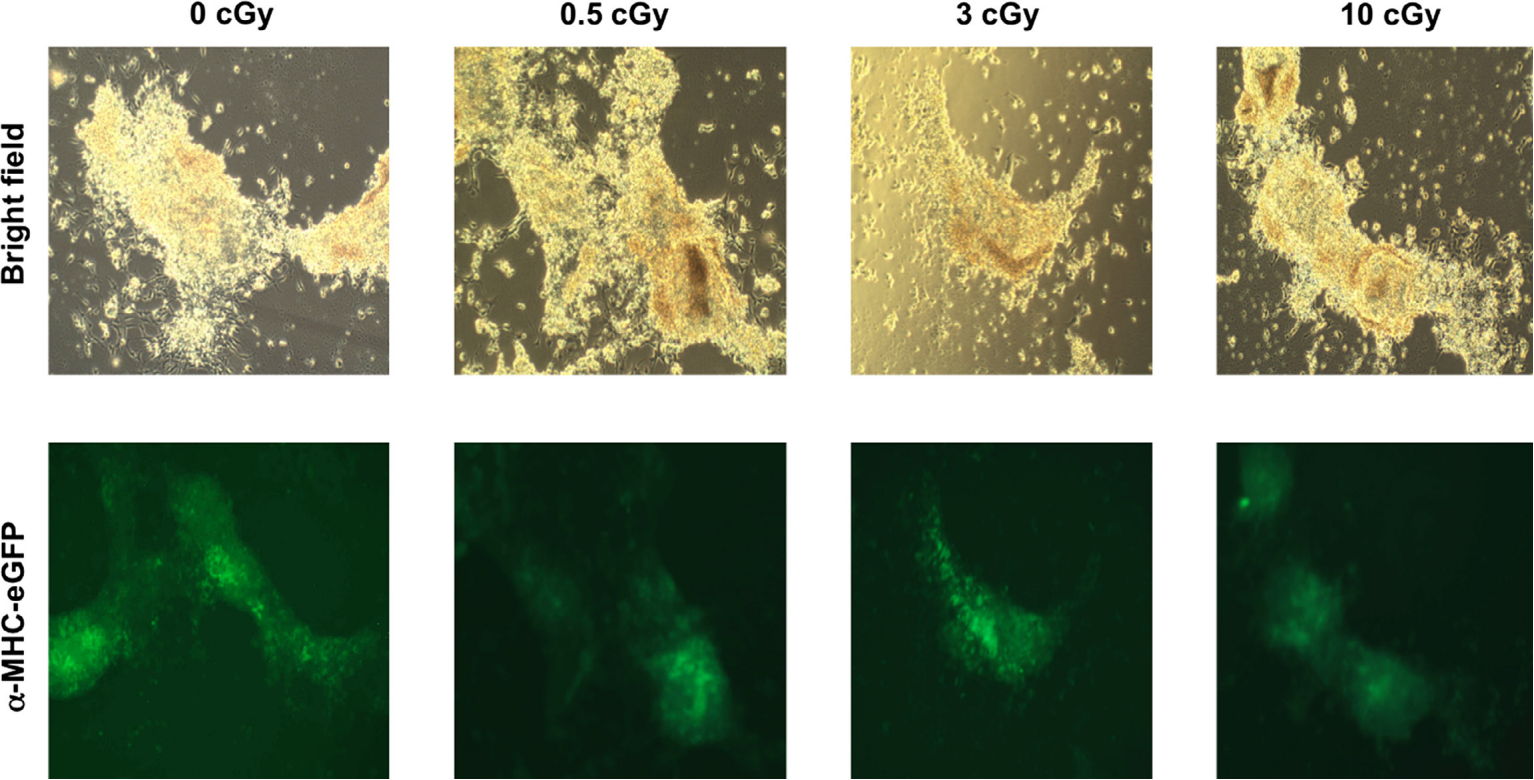
Alpha‐particle irradiation does not affect the onset of cardiac differentiation. HiPSC cell cultures maintained on Mylar films were irradiated with mean doses of 0.5, 3, and 10 cGy, transferred, and subjected to CM differentiation. HiPSC‐CMs were harvested at day 12 and replated on Matrigel‐coated glass bottom chambers. Top row: bright field, bottom row: GFP fluorescence under the cardiac promoter *α*‐MHC (*n* = 9).

**Figure 6:**
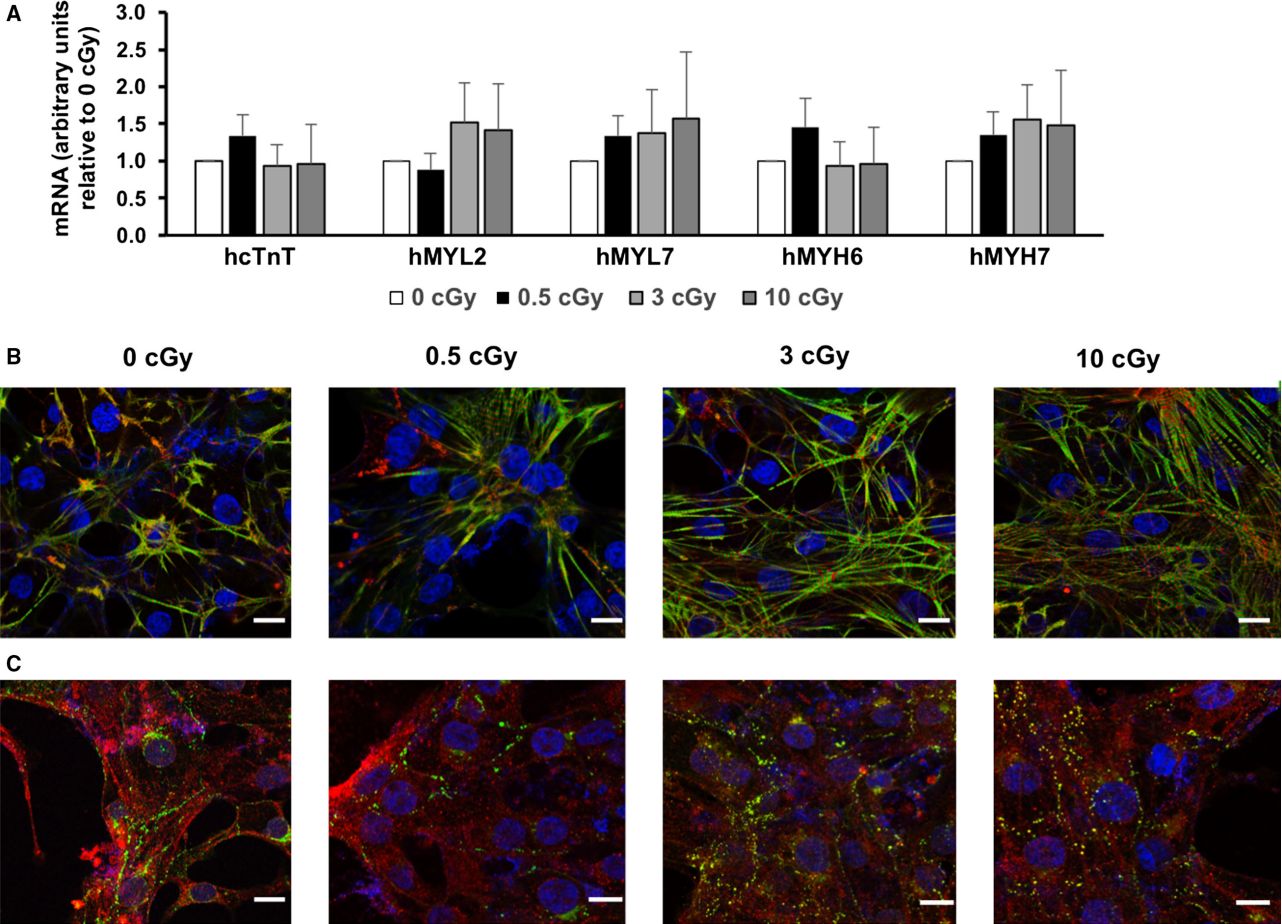
Alpha‐particle irradiation does not affect expression of cardiac markers. (A) hcTnT, hMYL2, hMYL7, hMYH6, hMYH7 expression were assessed by QRT‐PCR at day 12 in hiPSC‐CMs (*n* = 4). (B) Confocal images were obtained at day 20 from hiPSC‐CMs for hcTnT2 (green) + s‐actinin (red). Three biologically independent experiments were performed (*n* = 3). Scale bar = 10 *μ*m. (C) Confocal images were obtained at day 20 from hiPSC‐CMs for Cx43 (green) + MLC2v (red). Three biologically independent experiments were performed (*n* = 3). Scale bar = 10 *μ*m.

HiPSCs cultured on Mylar dishes were subjected to a mean dose of 0.5, 3, or 10 cGy of *α*‐particles (Figs. 3, 4, 5, 6). HiPSCs expressed the pluripotent marker Oct4 in irradiated as well as in control cells (Fig. 3A). Subsequently, the cells were replated and subjected to cardiac differentiation. No difference was observed in live cell count (Fig. 3B), dead cell count (Fig. 3C), cell proliferation (Fig. 3D), or total cell count (Fig. 3E) at various steps of cell differentiation, before and after addition of CHIR99021 or IWP‐2 (days −4, 0, 4, and 7; see Fig. 1A). However, cell number was markedly and dose dependently affected by irradiation at day 12 (Fig. 4). Cell beating started in all cultures at day 10 of differentiation (see Video S1), and all cultures expressed the *α*MHC‐GFP reporter gene (Fig. 5). Very few cells remained attached to the dish following the 10 cGy treatment, displaying fields with no cellularity (Fig. 4B). However, a minimal amount of cells was collected for each condition, which was sufficient to achieve further characterization. Contractile markers of cardiac differentiation were examined at days 12 and 20 (Fig. 6). hcTnT, hMYL2, hMYL7, hMYH6, and hMYH7 were expressed in irradiated and nonirradiated hiPSC‐CMs (Fig. 6A). There was a tendency for increased expression of contractile markers in the irradiated cells, although the difference was not statistically significant (Fig. 6A). Confocal images of irradiated and nonirradiated hiPS‐CMs at differentiation day 20 were also obtained. There was no difference in the expression of hcTnT2 and s‐actinin between conditions (Fig. 6B). Other cardiac markers, such as Cx43 and MLC2v, revealed no difference in expression and localization either (Fig. 6C). Taken together, *α*‐particle irradiation of hiPSC‐CMs leads to diminished cell number of differentiated cardiomyocytes, although the process of differentiation proceeds unaffected.

### Alpha‐particle irradiation increases the arrythmogenicity potential in hiPSC‐CMs

**Figure 7:**
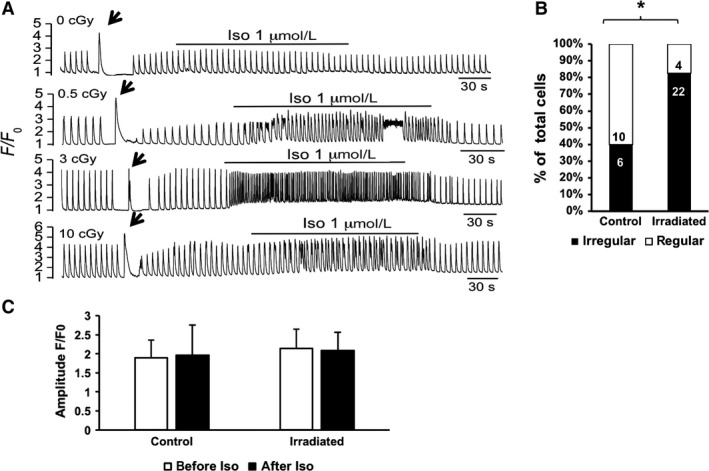
Rhythm disturbances are exacerbated in irradiated hiPSC‐CMs challenged with isoproterenol. (A) Representative calcium traces for nonirradiated control and irradiated hiPSC‐CM. Horizontal bar indicates isoproterenol (Iso, 1 *μ*mol/L) application in each panel. The arrow indicates caffeine‐induced calcium release from SR in each panel. (B) Quantification of % of cells showing Ca waves/spontaneous Ca transients in nonirradiated control and hiPS‐CM originating from irradiated cultures in response to Iso treatment. The numbers within the bars represent the sample size. Because every irradiation condition (0.5, 3, and 10 cGy) led to similar arrhythmic patterns, cells originating from irradiated cultures were pooled. Total number of cells analyzed: 16 nonirradiated cells and 26 cells originating from irradiated cultures. Total number of differentiation events: 4. *P < 0.01 with Fisher's exact test. (C) Quantification of the Ca^2+^ transient amplitude of irradiated and nonirradiated cells, before and after Iso. Total number of cells analyzed: 16 nonirradiated cells and 16 cells originating from irradiated cultures. Total number of differentiation events: 4. *P* > 0.05 with Fisher's exact test.

Intracellular Ca^2+^ transients were measured to access the functionality of the differentiated cells at days 20–30 after the cardiac differentiation of hiPSC (Fig. 7). Regular Ca^2+^ transients were elicited by field electrical stimulations at 0.2 Hz. *β*‐Adrenergic stimulation has been known to cause Ca^2+^ overload and oxidative stress. Although no significant increase in Ca^2+^ transient amplitude was observed (Fig. 7C) in any of the groups after treatment with 1 *μ*mol/L isoproterenol (Iso, a *β*1 adrenergic agonist), the incidence of spontaneous Ca^2+^ waves/releases was more frequent in the irradiated groups than in the nonirradiated control groups (representative tracings in Fig. 7A and quantification on Fig. 7B), suggesting that *α*‐particle irradiation may increase the susceptibility to arrhythmogenesis under adrenergic stress condition. Exposure to caffeine (10 mmol/L) caused comparable Ca^2+^ release from the SR among all the experimental groups (as indicated by the arrows in Fig. 7A).

### Mitochondrial stress and biogenesis markers

**Figure 8:**
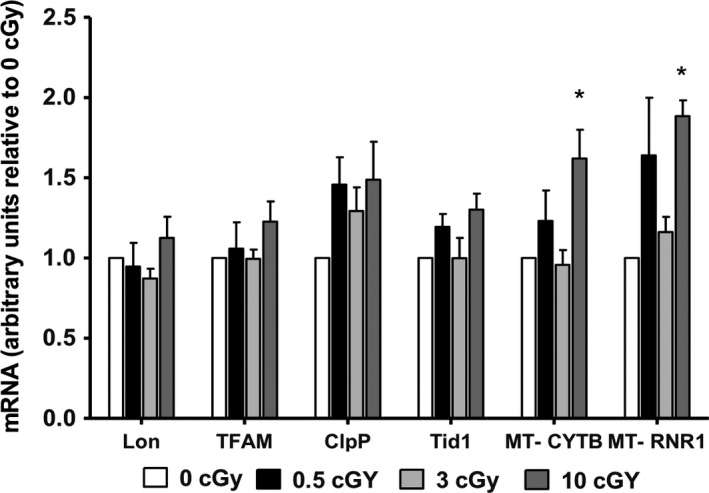
Alpha‐particle radiation upregulates mtDNA‐encoded transcript levels. Relative fold change in the Lon, TFAM, ClpP, Tid1, and mtDNA‐encoded MT‐ CYB and MT‐RNR1 transcripts in hiPSC‐CMs 12 days after they were differentiated from *α*‐particle‐irradiated hiPSCs (0.5, 3, and 10 cGy) (*n* = 5–6). *P* < 0.05 is considered as significant. Values are expressed as relative fold change ± SEM.

Ionizing radiation is a well‐characterized oxidizing agent. Its absorption results in both excitations and ionizations leading to production of free radicals (Azzam et al. 2012). In addition, it activates oxidases and nitric oxide synthases leading to the generation of reactive oxygen and nitrogen species (ROS/RNS) that can attack critical molecules in the cell (Azzam et al. 2012). Although ~60 ROS per nanogram of tissue are generated within less than a microsecond from a hit caused by ^137^Cs *γ* rays, ~2000 ROS are generated from a 3.2 MeV *α*‐particle traversal, which corresponds to a ROS concentration of ~19 nmol/L in the nucleus (reviewed in Azzam et al. 2012). Previous work has shown that ionizing radiation increases mitochondrial respiration and ATP production in A549 human lung adenocarcinoma cells (Yamamori et al. 2012), and that TFAM is upregulated by *α*‐particle radiation in A549 cells (Yu et al. 2013). After 12 days of differentiation, exposure of hiPSCs to 10 cGy significantly upregulated MT‐CYB and MT‐RNR1 transcript levels by 60% (*P *< 0.05) compared to sham‐irradiated cells (Fig. 8). Although a slight upregulation of Lon, ClpP, Tid1, and TFAM was noted at a dose of 10 cGy, this increase was not significant.

## Discussion

Human exposure to ionizing radiation likely impacts the stem cell capability to faithfully accomplish tissue development. However, it becomes challenging to identify a reliable in vitro model to study tissue differentiation and the consequences of exposure to low levels of densely ionizing particulate radiations that humans may be subjected to during radiotherapy or occupational exposures, especially during the first stages of life. To begin to address questions regarding radiation exposure on human cardiac differentiation, we developed a model of *α*‐particle radiation on hiPSCs. These irradiated cells were subsequently differentiated into beating CMs. Thus, this model provides an experimental system for investigating the deleterious effects of radiation exposure, or the adaptive responses elicited to mitigate such exposure, which influence the pathogenesis of human congenital heart disease.

We found that following exposure of the stem cells to even such low mean doses of *α*‐particles, the total number of differentiated CMs decreased in a dose‐dependent manner. However, the same exposure to ionizing radiation did not appear to affect the expression of pluripotent markers of the hiPSCs and did not alter cell number, cell death, or cell proliferation of hiPSCs at intermediate time points of differentiation. Further studies will help to determine if the low numbers of differentiated CMs result from a decrease in proliferation, an increase in cell death, or the inability of these cells to remain attached to the dish, particularly when they start beating. The latter effects may arise from the direct effects of the *α*‐particles or may be due to the spread of harmful effects from directly irradiated cells to bystander cells in their vicinity. Interestingly, at a mean absorbed dose of 0.5 cGy, only ~3% in the exposed population would receive an *α*‐particle traversal through the nucleus (Gonon et al. 2013).

The mean doses applied to the cells highlight some limitations. At the low mean doses used in this study, the differentiation process does not appear to be affected, even at the highest dose of 10 cGy. This has been seen at the level of contractile markers, onset of appearance of beating, GFP fluorescence, and the presence of Cx43 – a critical component of the CM gap junction. However, some aspects of the functionality of the cells seem to be impaired, evidenced through higher propensity of spontaneous Ca^2+^ releases upon isoproterenol challenge in the irradiated groups. Previous studies have suggested both Ca^2+^ overload and elevated oxidative stress level (increased ROS levels) are required for Iso‐induced high incidence of Ca^2+^ waves (Zhao et al. 2015). These results may provide a potential link between cardiac rhythm dysfunction and exposure to *α*‐particles. Further studies will be carried out to identify the underlying molecular mechanisms that govern radiation‐induced arrhythmogenesis in hiPSC‐CMs, and to determine how cellular exposure to *α*‐particle radiation affects remodeling of Ca‐handling proteins and oxidative stress‐responsive systems. Another limitation is the lack of irradiation studies conducted directly on differentiated cardiomyocytes. While our model will have implications for human embryogenesis, and consequently for congenital heart disease, the irradiation of differentiated cells may be more relevant to adult events and even aging processes of heart failure.

Some of the parameters that were affected may converge at calcium handling, which could be driven by an increase in ROS levels, a common deleterious consequence of exposure to ionizing radiation likely affecting metabolic activity (Azzam et al. 2012). These observations point to mitochondria as critical players in such processes, as they are major sites for metabolic ROS generation and make up ~40% of cardiomyocyte cell volume. Although the transcript levels of the four important mitochondrial stress response proteins Lon, ClpP, Tid1, and TFAM were found slightly upregulated 12 days postirradiation, the significant upregulation of the MT‐CYB and MT‐RNR1 transcripts, which are required for the electron transport chain and mitochondrial protein synthesis, respectively, suggested that cellular exposure to *α*‐particles has a sustained influence on mitochondrial function. Studies have shown that in transformed human small airway epithelial cells, *α* radiation induces mtDNA damage and also increases mtDNA copy number as well as OXPHOS (Zhang et al. 2013). The increased expression of MT‐CYB and MT‐RNR1 observed in *α*‐particle‐irradiated hiPSC‐CMs may represent a compensatory mechanism to upregulate ATP synthesis by OXPHOS. Although the transcript levels of Lon, ClpP, Tid1, and TFAM are slightly upregulated by *α*‐particle radiation, their protein levels and activity in response to ionizing irradiation may be significantly altered. Future studies are required to address these possibilities and to also determine whether *α*‐particles cause changes in calcium flux, which impact the differentiation of hiPSCs into cardiac myocytes.

Ionizing radiation is either electromagnetic or particulate. Although X‐rays and *γ* rays are electromagnetic radiation, energetic electrons, protons, neutrons, *α*‐particles, and heavy charged particles are different forms of particulate radiation (Hall and Giaccia 2006). While X‐rays and *γ* rays are sparsely ionizing radiations, therapeutic *α*‐particles or energetic heavy ions are densely ionizing (Hall and Giaccia 2006). Although the focus of this study is on the effects of *α*‐particles on cardiomyocyte differentiation from human pluripotent stem cells, the knowledge gained is relevant to other densely ionizing radiations such as energetic heavy ions used in cancer therapy or to which astronauts may be exposed during prolonged travel in deep space: *α*‐particles and energetic heavy ions share many biophysical characteristics (Li et al. 2014). Our study on human cells expands and complements recent studies on cardiac differentiation by therapeutic X‐rays and energetic carbon ions in mouse embryonic stem cells, which show a likely impact on murine cardiac differentiation (Helm et al. 2016).

We use *α*‐particles as a surrogate to examine the biological effects of densely ionizing radiations on cardiac differentiation. There is also a great interest in using *α*‐particle emitters for therapeutic purposes (Dahle et al. 2011; Baidoo et al. 2013). Alpha‐particle emitting radionuclides conjugated to monoclonal antibodies have long been advocated for the treatment of various cancers (Bloomer et al. 1981; Nilsson et al. 2005; Zalutsky et al. 2007; Behling et al. 2016). Furthermore, *α*‐particle vascular brachytherapy has been considered in the treatment of in‐stent restenosis (Mehdizadeh et al. 2009). This interest emanates from the biophysical properties of *α*‐particle radiation. Energy deposition by *α*‐particles with energies in the range of 2–10 MeV is densely structured along short linear tracks (Nikjoo et al. 2001). Furthermore, an enhanced rate of energy loss occurs at the end of the particle track (Allen et al. 2011). Such patterns of energy deposition in tissues render these particles highly effective per unit of absorbed dose at killing the cells they traverse (Hall and Giaccia 2006). However, off‐target effects may occur (Azzam et al. 2003). For example, intravenous administration of *α*‐particle emitters will unavoidably result in irradiation, albeit with low mean absorbed doses, of heart and vascular tissues. An understanding of the consequences of irradiation in general on cardiac differentiation is particularly relevant in case of pregnant women, when the fetus may be exposed, and where there is a paucity of data (Helm et al. 2016).

An excess risk of developing cardiovascular disease is thought to have occurred in the A‐bomb survivors even following low dose exposure (<100 mSv) (Little 2009, 2013). Therefore, understanding the biological effects of exposure of pluripotent stem cells to *α*‐particles and other types of radiation is relevant to radiation protection as well as to the development of countermeasures that may alleviate harmful side effects of radiotherapy with densely ionizing radiations.

## Conflict of Interest

None declared.

## Data Accessibility

## Supporting information




**Video S1–S3.** Beating hiPSC‐CMs (0, 0.5, and 3 cGy).
**Figure S1.** Analysis of hiPSCs. (A–D) HiPSCs were cultured on Matrigel‐coated cover slips in 60 mm dishes in mTeSR1 for 2 days. (A) Bright field image. (B) TRA‐1‐60 live staining (green). (C) Oct 3/4 staining (green). (D) hNanog staining (green). Blue: DAPI counterstain. Scale bar = 100 *μ*m.
