# Supplementary figures and images for: Effect of densely ionizing radiation on cardiomyocyte differentiation from human‐induced pluripotent stem cells
Source: Physiol Rep. 2017 Aug 11;5(15):e13308. doi: 10.14814/phy2.13308 (PMC5555881; doi:10.14814/phy2.13308)

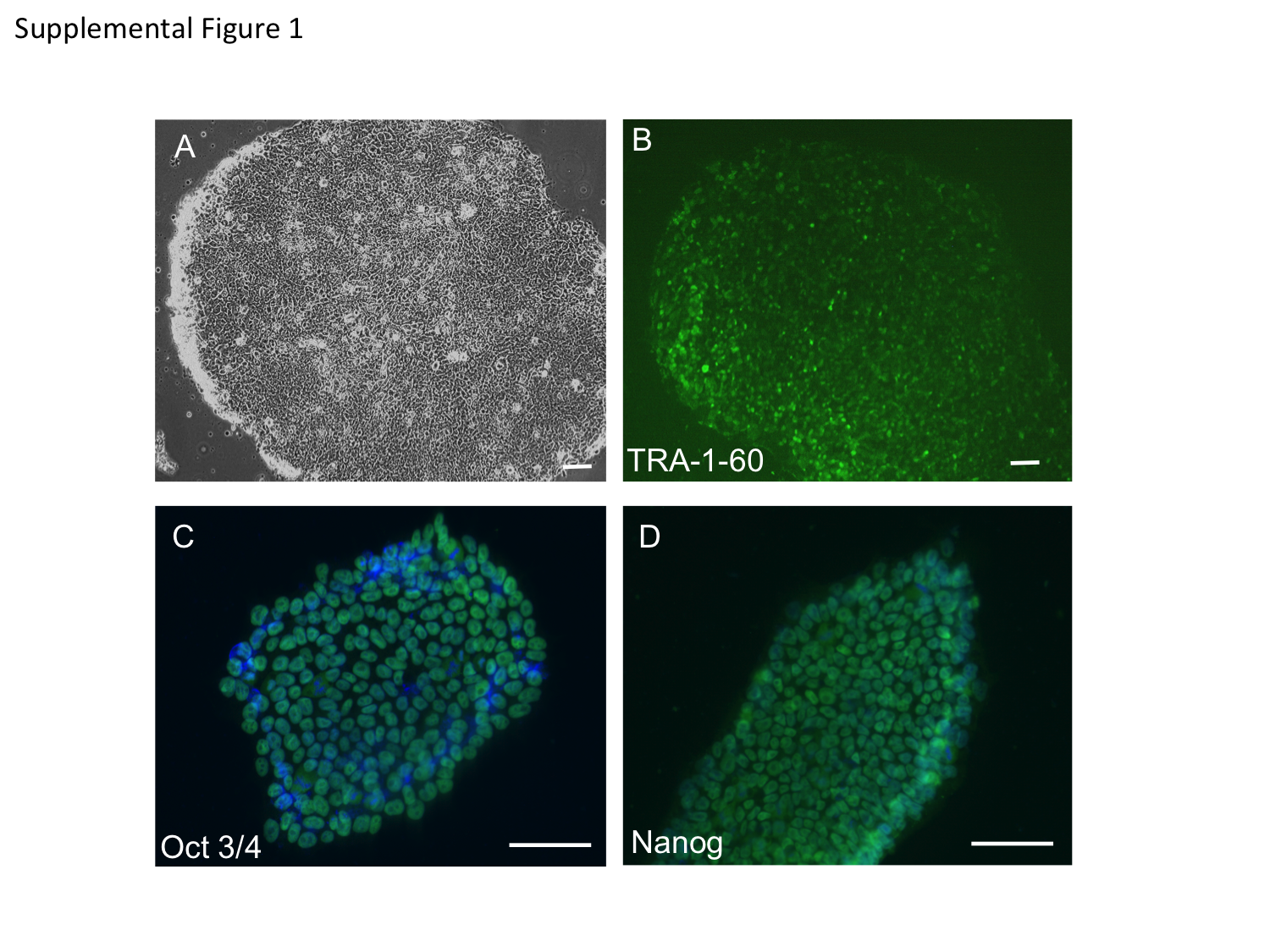

Supplement: Supplementary file 2 [file PHY2-5-e13308-s002.zip › Baljinnyam_Supplemental_Figure1_050117.tif]
